# Immunomodulatory biomaterials and regenerative immunology

**DOI:** 10.4155/fsoa-2016-0060

**Published:** 2016-09-29

**Authors:** Nihal Engin Vrana

**Affiliations:** 1 Protip Medical, 8 Place de l'Hopital, 67000 Strasbourg, France; 2 INSERM UMR 1121, 11 Rue Humann, 67000 Strasbourg, France

**Keywords:** biomaterials, immunology, immunomodulation, macrophages, regenerative medicine

Organ damage and loss remain important clinical problems, where the current gold standard is transplantation. One of the main problems with transplants, even when they are successful, is the need to use immunosuppressants (IS) for preventing acute rejection. The use of IS entails several side effects such as increased risk of tumor formation, higher susceptibility to infections and IS-related toxicity. Even though in certain cases, such as liver transplants, weaning off the IS has been achieved [1], in many transplant scenarios the use of IS is indispensable. This aspect was one of the initial promises of tissue engineering as the use of autologous cells and biocompatible biomaterials would render the use of IS unnecessary.

In order to prevent rejection, a strict donor–recipient match is necessary and although a high level of efficacy has been achieved with transplant recipients (>95% 1-year survival rate), the increasing number of patients on the donor waiting lists without a corresponding increase in the number of donated organs demonstrates the need for an alternative source of replacement organs. Moreover, beyond the donor–recipient matching, the level of long-term success will be dependent on the demographic data pertaining to donor and the donor's medical history together with the reason of death. For example, donors for whom the reason of death was cerebrovascular disease have been shown to be more prone to induce rejection [2]. Furthermore, even though the survival rates are high, the deterioration of the transplant is not completely evitable. T-cell mediated scarring of the allograft or antibody-mediated processes will still be active.

Immunomodulation methods beyond systemic immunosuppression have been under development. Such technologies include donor regulatory T-cell therapy for promoting tolerance, antibody-based approaches for suppression of alloreactive T cells, allogenic antigen presentation methods during autologous cell debris scavenging to induce tolerance or low-dose IL-2 application for increasing host-regulatory T-cell numbers [3,4]. The use of biomaterials as immunomodulatory agents started with the encapsulation of allogenic Langerhans islets for treatment of diabetes using materials with low immunogenicity such as alginate. This provides an active barrier between the immune cells and antibodies of the host, and the metabolically active implanted allogenic cells. In a similar vein, biomaterial-based controlled delivery systems can achieve local control of immune response, hence circumventing most of the side effects of systemic immunomodulation [5].

In the context of regenerative medicine, these methods can provide means to utilize allogenic cell sources rather than autologous cells for patients with congenital diseases or with compromised cell populations. Moreover, coupling of immunomodulatory agents directly to biomaterials to render them immunomodulatory biomaterials [6] will help the development of highly remodelable scaffolds *in vivo* with regenerative properties beyond the inherent immunomodulatory activities of systems such as decellularized tissues [7].

Tissue engineering and regenerative medicine have come a long way in solving problems pertaining to organ/tissue loss and damage. In the last 10 years, more and more cases of clinical implementation of tissue engineering have been published with short- and long-term successes [8]. Moreover, the advances in tissue engineering have also led to development of complex 3D *in vitro* artificial tissue and organ systems that are being actively improved for replacement of animal experiments and also as more relevant models for drug and pharmacological tests [9]. These successes give hope for design and implementation of more complex tissues. For development of fully functional engineered tissues incorporation of immune cells or immunomodulatory elements might have significant benefits [10].

Even though tissues can be defined in a manner that emphasizes their specific function, barring certain exceptions, the presence of the components of three other systems of the body (namely, innervations, incoming and outgoing vasculature and resident immune cells) are common for all tissues. Because of the complexity of achieving a fully developed tissue, tissue engineering research has generally focused on the production of tissue-like structures containing the main functional cells of a tissue (such as chondrocytes for cartilage or osteoblasts for bone) where certain tissue-specific characteristics are taken as a marker of tissue maturation. Even though successful differentiation and microtissue formation in these settings can be achieved *in vitro*, for actual clinically relevant size defects, the necessity of integration with the host vasculature has been realized. In order to facilitate the integration, several different methodologies have been devised such as sacrificial microfluidic channels in scaffolds for improving capillary in-growth; addition of chemoattractants and growth factors to induce angiogenesis toward the implanted artificial tissue; and incorporation of vascular endothelial cells for prevascularization [11]. Co-culture of endothelial cells with other cell types generally induced not only capillary sprouting but also a synergistic interaction between the two cell types that contribute to the maturation of the engineered tissue.

The next in line in the sophistication of engineered tissues can be the inclusion of the immune system component. Nearly all tissues have resident macrophage populations which has been shown to be an important factor in tissue homeostasis and healing upon injury [12]. Recently, there has been a growing focus on the control over innate immune response in the microenvironment of implanted materials particularly through well-established macrophage polarization pathways that have been shown to have a crucial role in vascularization of implanted scaffolds [13]. Immunoassisted tissue engineering approaches can harness the ability of innate immune cells to resolve inflammation and promote regeneration and healing. This can be achieved by exploiting the phenotypic plasticity of immune cells either via controlled delivery of specific phenotype inducing cytokines [14] or direct co-delivery of phenotype controlled immune cells together with the cells relevant to the target organ function.

A new focus on establishing a cross-talk with the host immune system, rather than trying to evade it, could pave the way for more functional and fast-integrating artificial tissues. Concomitant use of new developments in temporal control of multiple growth factor/cytokine delivery; advanced bottom-up assembly methods of engineered tissues such as robotic assembly [15]; use of bioactive miRNAs within scaffolds; and micro/nanoscale topographical and chemical control of scaffold features [16] for inducing anti- or proinflammatory immune cell phenotypes would provide the tools for engineering multicellular organs and establishing *in vitro* organoids that faithfully model physiological conditions with immune system components. These efforts would bring forth the aspects of ‘regenerative immunology’ in regenerative medicine.
